# The plastid-nucleus localized DNA-binding protein WHIRLY1 is required for acclimation of barley leaves to high light

**DOI:** 10.1007/s00425-022-03854-x

**Published:** 2022-03-13

**Authors:** Monireh Saeid Nia, Urska Repnik, Karin Krupinska, Wolfgang Bilger

**Affiliations:** 1 Institute of Botany, Christian-Albrechts-University, Kiel, Germany; 2 Central Microscopy, Department of Biology, Christian-Albrechts-University, Kiel, Germany

**Keywords:** WHIRLY1, High light acclimation, Photosynthetic capacity, Carboxylation efficiency, RubisCO abundance, Leaf thickness, Leaf mass per area (LMA)

## Abstract

**Main conclusion:**

**In accordance with a key role of WHIRLY1 in light-acclimation mechanisms, typical features of acclimation to high light, including photosynthesis and leaf morphology, are compromised in WHIRLY1 deficient plants.**

**Abstract:**

Acclimation to the environment requires efficient communication between chloroplasts and the nucleus. Previous studies indicated that the plastid-nucleus located WHIRLY1 protein is required for the communication between plastids and the nucleus in situations of high light exposure. To investigate the consequences of WHIRLY1 deficiency on the light acclimation of photosynthesis and leaf anatomy, transgenic barley plants with an RNAi-mediated knockdown of *HvWHIRLY1* were compared to wild-type plants when growing at low and high irradiance. While wild-type plants showed the typical light acclimation responses, i.e. higher photosynthetic capacity and thicker leaves, the WHIRLY1 deficient plants were not able to respond to differences in irradiance. The results revealed a systemic role of WHIRLY1 in light acclimation by coordinating responses at the level of the chloroplast and the level of leaf morphology.

**Supplementary Information:**

The online version contains supplementary material available at 10.1007/s00425-022-03854-x.

## Introduction

Due to their sessile way of life, plants continuously encounter dynamic environmental conditions and their survival depends on appropriate responses to these variations (Nelson and Ben-Shem 2004; Dietzel and Pfannschmidt 2008). Chloroplasts are important sensors of environmental changes (Kleine et al. 2021) such as high light (Munné-Bosch 2019). Compounds produced by chloroplasts act as retrograde signals regulating nuclear gene expression to allow for acclimation to the environment (Chan et al. 2016; Pfannschmidt et al.^2020^). Plants may encounter stress-induced damage, if the acclimation is not achieved (Dietz 2015).

Acclimation to the environment involves mechanisms ensuring efficient photosynthesis by adjustments in the composition of the photosynthetic apparatus consisting of plastid and nucleus-encoded proteins (Björkman 1981; Race et al. 1999; Allen et al. 2011) as well as by changes in the morphology of leaves (Givnish 1988; Terashima et al. 2006; Poorter et al. 2009; 2019) such as increased leaf thickness and leaf mass per area (LMA).

Plants acclimated to high irradiance have a higher photosynthetic capacity, defined as leaf area-based light and CO_2_ saturated rate of CO_2_-fixation, than low light acclimated plants (Boardman et al. 1975; Lichtenthaler et al. 2007; Athanasiou et al. 2010). Acclimation involves changes in the abundance or organization of protein complexes in thylakoid membranes (Zivcak et al. 2014), a higher rate of electron transport (Leong and Anderson 1984a; Evans, 1987), higher levels of photosystem II (PSII), cytochrome b/f complex (Leong and Anderson 1984a, b), together with higher photophosphorylation rate (Murchie and Horton 1997) as well as a greater concentration of different components of the Calvin–Benson cycle, especially ribulose-1,5-bisphosphate carboxylase/oxygenase (RubisCO) (Leong and Anderson 1984a, b; Foyer et al. 2012; Vialet-Chabrand et al. 2017; Poorter et al. 2019).

The acclimation of photosynthesis in response to changes in the environment is based on massive changes in gene expression. The majority of about 3000 different plastid proteins is nuclear-encoded. Their transcription during high light is controlled by various signal compounds produced by chloroplasts such as ROS, cyclocitral, methylerythriol cyclodiphosphate (MEcPP), and phosphoadenosine 5′-phosphate (PAP) (Pfannschmidt et al. 2020). In recent years, it became obvious that also dually located plastid-nucleus proteins such as the DNA binding protein WHIRLY1 are involved in the communication between chloroplasts and the nuclear genomes (Bobik and Burch-Smith 2015; Krupinska et al. 2020). In transplastomic tobacco plants, WHIRLY1 was shown to translocate from chloroplasts to the nucleus (Isemer et al. 2012). It has been hypothesized that the translocation of WHIRLY1 from chloroplasts to the nucleus is induced upon stress-associated redox changes in the photosynthetic apparatus (Foyer et al. 2014). To investigate its role in plastid-nucleus communication, barley plants with a very strongly reduced level of WHIRLY1 were prepared by RNAi-mediated knockdown of *HvWHIRLY1* (Krupinska et al. 2014). Leaves of one of these lines, i.e. RNAi-WHIRLY1-7, contain only about 1–5% of the WHIRLY1 amount of wild-type leaves (Krupinska et al. 2014). These plants showed retardation of all phases of leaf development including senescence. While high irradiance was able to promote senescence of wild-type leaves, it barely affected the senescence of the WHIRLY1 deficient plants (Kucharewicz et al. 2017). When the WHIRLY1 deficient plants were grown in continuous light of different irradiances, their leaves showed severe symptoms of oxidative stress (Swida-Barteczka et al. 2018) such as bleaching, reduction of PSII efficiency, and the accumulation of ROS. The severity of these symptoms, that are typical for oxidative stress resulting from excess excitation energy in chloroplasts, correlated with the amount of residual WHIRLY1. The phenotype of the WHIRLY1 deficient leaves suggested that they are impaired in acclimation to light.

This study aimed to investigate the involvement of WHIRLY1 in retrograde signaling during high light acclimation of the photosynthetic apparatus in more detail. For this purpose, photosynthetic parameters such as CO_2_ assimilation rate, RubisCO content, and its in vivo activity were characterized in both WHIRLY1 deficient and wild-type leaves at different irradiances and different developmental stages. In addition, acclimation was investigated at the level of leaf morphology. The results revealed that acclimation of the photosynthetic apparatus as well as of leaf morphology require a high abundance of the WHIRLY1 protein.

## Materials and methods

### Plant material and growth conditions

Grains of *Hordeum vulgare* L., cv. “Golden Promise” wild-type (WT) and the WHIRLY1 deficient plants prepared by RNAi-mediated knockdown of HvWHIRLY1 (W1) (Krupinska et al. 2014) were sown in soil (Einheitserde ED73, Einheitswerk Werner Tantau, Uetersen, Germany). Pots were kept in darkness at 6 °C for three days to synchronize germination and were then transferred to climate chambers (Johnson Control, Mannheim, Germany) equipped with ceramic metal halide lamps (CMT360LS WBH EYE Iwasaki Electric Co., Japan). Plants were grown in a light/dark regime of 16/8 h, and a temperature of 21 °C and ca. 60% air humidity. Photosynthetic photon flux densities incident on the leaf surface were either 350–500 µmol m^−2^ s^−1^ (high light, HL) or 40–70 µmol m^−2^ s^−1^ (low light, LL), which corresponded to horizontal irradiances of 1000 or 150 µmol m^−2^ s^−1^. Irradiances incident on the adaxial and abaxial sides of the leaves were measured using a quantum sensor (Li-185 A, Li-Cor Biosciences, Lincoln, NE, USA). This was done for every individual primary leaf. The area between 1.5 and 3 cm below the tip of the primary leaves was used for all measurements. Ten-day-old primary leaves were used for most measurements if not otherwise mentioned.

### Gas exchange measurements

Light dependences of the CO_2_ assimilation rate (*A*) at the constant CO_2_ concentration of 1500 ppm and *A*/*C*_i_ curves at a PPFD of either 1000 µmol m^−2^ s^−1^ or 1500 µmol m^−2^ s^−1^ (selected based on preliminary measurements of photosynthetic light dependencies with the aim to assure light saturation but to avoid photoinhibitory damage) were measured by a portable Gas Exchange Fluorescence System GFS-3000 (Heinz Walz GmbH, Effeltrich, Germany). The instrument was set up at 750 µmol min^−1^ flow rate, a cuvette temperature of 21 °C, and 60% relative humidity. Attached primary leaves of both genotypes grown either in HL or LL were measured at different developmental stages from day 10 until day 19. A stable photosynthesis rate was induced at 380 ppm CO_2_ and a PPFD of 100 µmol m^−2^ s^−1^ for about 10 min, followed by a stepwise increase in irradiance until light saturation of photosynthesis was reached. Afterwards, CO_2_ was reduced from 380 to 50 ppm in five steps. The in vivo activity of RubisCO can be indicated by the carboxylation efficiency (CE) of RubisCO, and calculated as the initial slope of the *A*/*C*_i_ curve determined at an internal CO_2_ concentration (*C*_i_) below 200 ppm (von Caemmerer and Farquhar 1981; Cheng and Fuchigami 2000). CO_2_ concentration was set back to 380 ppm and increased stepwise to a maximum of 2000 ppm to measure the photosynthetic capacity (*P*_max_) which is defined here as the light and CO_2_ saturated rate of CO_2_-fixation per leaf area (Oguchi et al. 2003; Athanasiou et al. 2010; Townsend et al. 2018) and was measured at light saturation in the presence of 2000 ppm CO_2_.

The leaf segment area included in the cuvette was determined from a photograph using ImageJ software (US National Institutes of Health, Bethesda, Maryland, USA) to correct the photosynthetic rate according to the equations provided in the manual (Walz GmbH). Errors that might be caused by CO_2_ absorption in the cuvette at very low CO_2_ concentrations were corrected by measuring the same parameters, as done for leaves, in the absence of a leaf according to Long and Bernacchi (2003).

Leaf segments used for gas exchange measurements were frozen in liquid nitrogen and kept in a freezer at − 80 °C to be later analysed for their RubisCO content (see the section on determination of RbcL abundances by SDS-PAGE).

### Non-invasive measurements of chlorophyll contents

Chlorophyll contents were measured non-invasively by a Dualex Scientific instrument (Force A, Paris, France). Measurements at three points between 1.5 and 3 cm below the tip in each leaf were averaged. Readings of the Dualex instrument were calibrated by extraction of leaf segments and determination of chlorophyll contents by HPLC (see below). The calibration function determined by linear regression was ${\text{Chl }}\left[ {{\text{nmol}}\;{\text{cm}}^{ - 2} } \right] = 1.0381 \times {\text{Dualex}}\;{\text{reading}} + 8.7495$.

### HPLC analysis of chlorophyll contents

Exactly 1 cm long leaf segments from the area between 1.5 and 3 cm below the tip were cut in the climate chamber under growth irradiance. After a quick determination of the segments’ widths, they were immediately frozen in liquid nitrogen and stored at − 80 °C. To extract pigments, frozen segments were ground with 0.9 ml 80% (v/v) acetone (prepared with an aqueous solution of 30 mM Tris-buffered at pH 7.8) and 5–6 glass beads in a Geno Grinder (Type 2000; SPEX CertiPrep, Munich, Germany). After centrifugation for six minutes at 12,000 rpm at 4 °C (Kendro Biofuge fresco, Osterode, Germany), the pellets were extracted two more times with 0.3 ml of pure acetone. Finally, 0.05 ml of the combined supernatants were used for pigment analysis using an Agilent 1100 HPLC system (Waldbronn, Germany). As described by Nichelmann et al. (2016), pigment separation was done using a Hypersil ODS-column (4.6 × 250 mm, 5 µm particle size, Thermo Fisher Scientific Inc., Waltham, U.S.A.) using a mobile phase consisting of a gradient from 25% solvent A (10 mM Tris buffer (Roth) at pH 7.8) to 100% solvent B (100% acetone (Roth)). Chl *a* and *b* were identified through their retention time and absorption spectra monitored by a photodiode array detector (Agilent). Chlorophyll *a* and *b* were externally calibrated using the equations of Porra et al. (1989). For further details, see Nichelmann et al. (2016).

### Determination of the amount of the large subunit of RubisCO (RbcL) by SDS-PAGE

Proteins were extracted from a pool of 3–6 leaf segments. SDS-PAGE was performed with samples adjusted to five different protein concentrations (20, 15, 8, 4, and 2 µg per lane) using 16% (w/v) polyacrylamide gels (Laemmli 1970). Gels were stained in colloidal Coomassie (Dyballa and Metzger 2009). To estimate the amount of RubisCO, gel photographs were analyzed by ImageJ software. Signal intensities of different bands were plotted against protein concentrations loaded on the gels. For the initial linear relationship between both parameters, a linear regression was calculated and its slope considered as the relative amount of RubisCO per protein. This was then related to leaf area by multiplication with the amount of total proteins per leaf area (relative content of RubisCO cm^−2^). All the values were normalized to the relative amount of RubisCO in low light-grown WT plants at day 10 (as 100%) in each independent experiment and accordingly, the final values are presented as % relative RubisCO cm^−2^.

### Leaf morphology

#### Leaf cross section

##### Resin embedding for morphological and ultrastructural analyses

A mid part of a primary foliage leaf was cut into several 2–3 mm broad transverse stripes and fixed with 1% glutaraldehyde in 200 mM Hepes, pH 7.4, initially under moderate vacuum pressure. For resin embedding, stripes were cut further into 2–3 mm broad longitudinal segments. These were post-fixed with 1% osmium tetroxide (Roth, Karlsruhe, Germany) in 1.5% aqueous potassium ferricyanide (Merck, Darmstadt, Germany) for 1 h on ice, followed by incubation with 2% aqueous uranyl acetate (Science Services, München, Germany) for 1.5 h at room temperature, protected from light. Tissue was dehydrated using a graded ethanol series (50–70–80–90–96–100–100%), each step for 15–30 min, followed by acetone (100%), 2 × 30 min, and then gradually infiltrated with epoxy resin diluted with acetone (25–50–75–100%), each step for minimum 12 h. Finally, tissue was flat embedded in silicone molds and resin was heat polymerised at 70 °C for 24 h. Sections were cut using a Leica UC7 ultramicrotome and Diatome diamond knives.

Semithin, 500-nm epon sections were transferred on water drops onto Superfrost Plus glass slides (Gerhard Menzel GmbH, Braunschweig, Germany), which were placed on a heated plate (90 °C) to allow water to evaporate and sections to adhere. Adhered sections were stained with Richardson’s solution (alkaline solution of azure II and methylene blue (Sigma-Aldrich)) for about 60 s at 90 °C and then washed with water. Stained sections were mounted with a Leica CV mount reagent, and imaged in a Zeiss Primostar upright microscope equipped with an Axiocam 105 color camera and the ZEN 3.2 software (Zeiss, Oberkochen, Germany), using 10× and 40× Plan-Achromat objectives.

#### Leaf thickness

Leaf thickness was determined in manual cross-sections by bright field microscopy (Microscope: Axiophot, Plan-Apochromat, 10×/0.45, Zeiss; camera: Olympus DP7, Olympus, Japan; image recorded by cell^F software version 5.1, Olympus) for two sections per sample and the average of these two measurements was used.

#### Leaf mass per area (LMA)

The LMA was calculated as the ratio between leaf dry mass and area. Leaf segments taken at 1.5–3 cm below the tip were scanned and the area determined using the Sigma Scan Pro 5 software (Systat Software, San José, CA). Afterwards, the leaf segments were dried in a laboratory oven at 60 °C for 24 h before dry weight was determined on a lab scale (AW-224, Sartorius, Germany).

### Transmission electron microscopy (TEM) for analysis of cytoplasm/cell volume

Ultra-thin, 80-nm epon sections were transferred onto formvar coated slot grids, and contrasted with saturated aqueous uranyl acetate for 10 min, followed by 0.2% lead citrate for 3 min. Sections were inspected in a Tecnai G2 Spirit BioTWIN transmission electron microscope (FEI, now Thermo Fisher Scientific), operated at 80 kV, and equipped with a LaB6 filament, an Eagle 4 k × 4 k CCD camera and a TIA software (both FEI, now Thermo Fisher Scientific).

The volume fraction of cytoplasm in mesophyll cells was determined by a stereological analysis of electron micrographs. Images were collected at 890 × magnification by systematic uniform random (SUR) sampling. In the Fiji software (Schindelin et al. 2012), 15 and 30 μm^2^ square test grids were used for point counting to estimate the area of the cytoplasm and of cell profiles, respectively. For each leaf, between 15 and 23 electron micrographs were analysed. The cytoplasm to cell volume fraction was calculated as a ratio between the total count of points over the cytoplasm and the total count of points over the reference area of cells. For each sample group, at least three leaves were analysed to obtain three estimates.

### Statistical analysis

For the statistical analysis, Sigmaplot 13 (Systat Software GmbH, Ekrath, Germany) or GraphPad PRISM (Prism 9 for Windows, version 9.2.0 (332), GraphPad software, San Diego, California USA) was used. One-way, two-way (with the factors of genotype and light conditions), or three-way ANOVA (with the factors of genotype, light conditions, and age) were used to analyze the data and the Holm–Sidak method was used for post hoc analysis. All the graphs were prepared using Sigmaplot 13 software.

## Results

### Photosynthetic gas exchange

#### Light dependency of CO_2_ assimilation

**Fig. 1:**
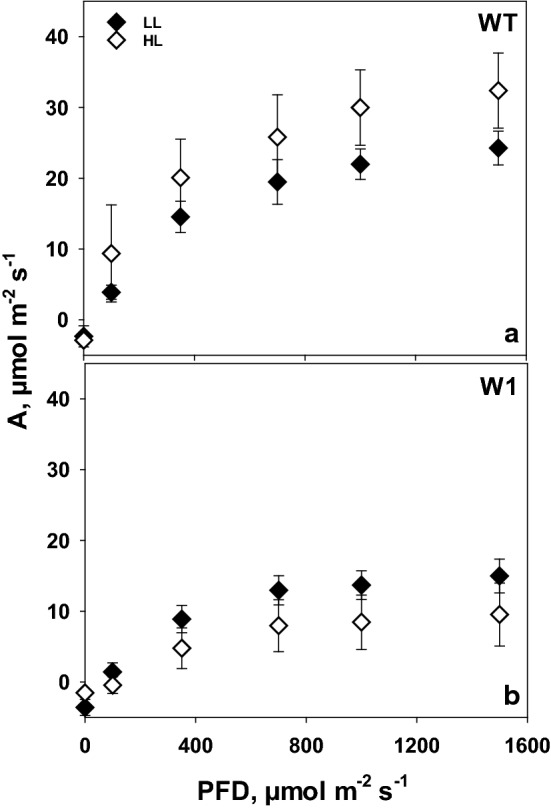
CO_2_ assimilation rate as a function of incident irradiance (PFD) measured in the presence of 380 ppm CO_2_ in LL (filled symbols) and HL (open symbols) grown plants for both WT (**a**) and W1 (**b**) at day 10 after sowing. Depicted values are means ± standard deviation of *n* = 9–15 leaves in total from three independent experiments each comprising 3–5 leaves

The CO_2_ assimilation rate (*A*) at saturating light (in the presence of 380 ppm CO_2_) in barley wild-type (WT) plants showed significantly (analyzed for the *A* values at maximum irradiance by two-way ANOVA, Tab. S1, *P* < 0.001) higher values for plants grown in high light in comparison to low light at day 10 (Fig. 1a).

While photosynthesis of 10-day-old wild-type plants showed significant responses to growth irradiance, WHIRLY1-deficient transgenic plants (W1) did not show acclimation and had even significantly (*P* = 0.008) lower rates of CO_2_ assimilation when grown under HL than LL (Fig. 1b). Both, in HL and LL, the CO_2_ assimilation rates of the WHIRLY1 deficient plants were significantly (*P* < 0.001) lower than those of the wild type (Fig. 1b).

#### ***A***/***C***_i_ curve

**Fig. 2:**
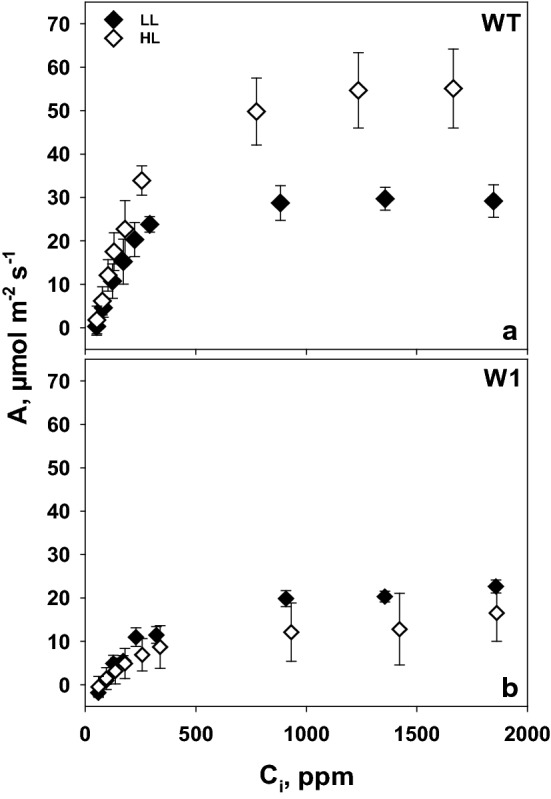
CO_2_ assimilation rate as a function of internal CO_2_ concentration (*C*_i_) measured in LL (filled symbols) and HL (open symbols) grown plants for both WT (**a**) and W1 (**b**) at day 10 with 1000–1500 µmol m^−2^ s^−1^ light as the saturating light. Depicted values are means ± standard deviation of *n* = 9–15 leaves in total from three independent experiments each comprising 3–5 leaves

To further analyze photosynthesis, CO_2_ assimilation rate was measured at saturating irradiance employing different CO_2_ concentrations. The increased assimilation rate in wild-type plants grown under high light in comparison to those from low light was especially obvious in the presence of a high CO_2_ concentration (Fig. 2a)_._ In the WHIRLY1 deficient plants, no acclimation of CO_2_ assimilation rate was detectable (Fig. 2b). High light-grown W1 plants showed even lower *A* than LL-grown plants, indicating potential photoinhibitory damage.

Besides the large difference in CO_2_ saturated assimilation rate, also the initial slope of the *A*/*C*_i_ curves, representing carboxylation efficiency (CE), showed a positive light acclimation in WT leaves and was clearly lower in W1 plants.

#### Non-invasive analysis of chlorophyll contents

**Fig. 3:**
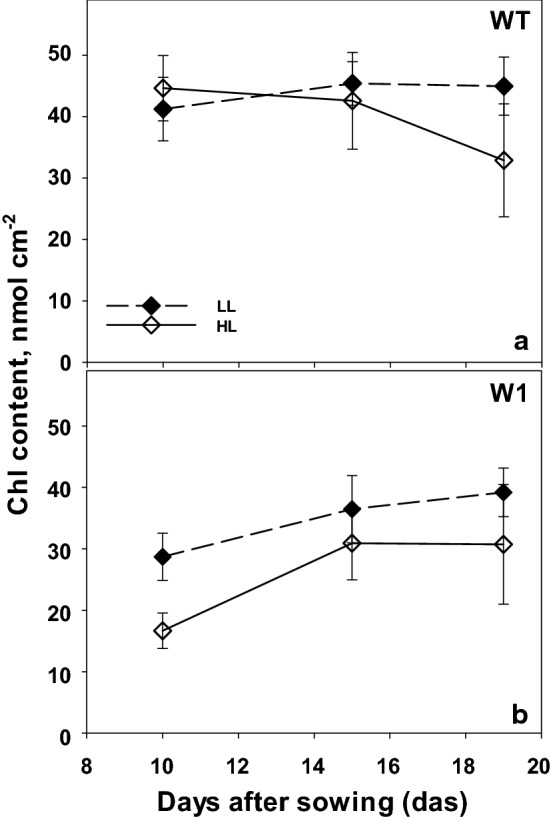
Chlorophyll content as a function of days after sowing in WT (**a**) and W1 (**b**) plants grown under low (LL, filled symbols) and high light (HL, open symbols). Depicted values are means ± standard deviation of *n* = 23–37 leaves in total from three independent experiments each comprising 7–13 leaves

WHIRLY1 deficient plants showed a delayed chloroplast development which was apparent in their chlorophyll contents, which were determined non-invasively (Fig. 3b). In total, 10-day-old WT plants did not show significant differences (Three-way ANOVA, Tab. S2, *P* = 0.289) in their leaf chlorophyll content when plants grown under LL and HL were compared (Fig. 3a). However, in contrast to the rather stable chlorophyll content of wild-type plants grown in low light over time (no statistical difference), chlorophyll contents of high light-grown wild-type plants decreased strongly from day 15 to day 19 (*P* < 0.0001).

In the W1 plants, chlorophyll content increased significantly (*P* < 0.0001, for both LL and HL plants) from day 10 to day 15 (Fig. 3b) which is in accordance with the reported delayed chloroplast development (Krupinska et al. 2019).

Three-way ANOVA analysis (Tab. S2) showed significant differences between WT and W1 plants grown under either low or high light at days 10 and 15 (*P* < 0.0001).

Because of the potential developmental influence on photosynthesis, all CO_2_ assimilation rates at different irradiances and CO_2_ concentrations were also calculated per chlorophyll content of 10-day-old primary leaves of WT and W1 plants (Supplementary data Fig. S1 and Fig. S2, respectively) and showed similar tendencies as the results calculated per leaf area (Figs. 1 and 2).

#### Photosynthetic capacity

To further investigate whether the delay in chloroplast development was responsible for the observed differences in photosynthesis, the photosynthetic capacity (*P*_max_) and carboxylation efficiency (CE) were also measured in 15- and 19-day-old WT and transgenic plants grown under low and high irradiances.

**Fig. 4:**
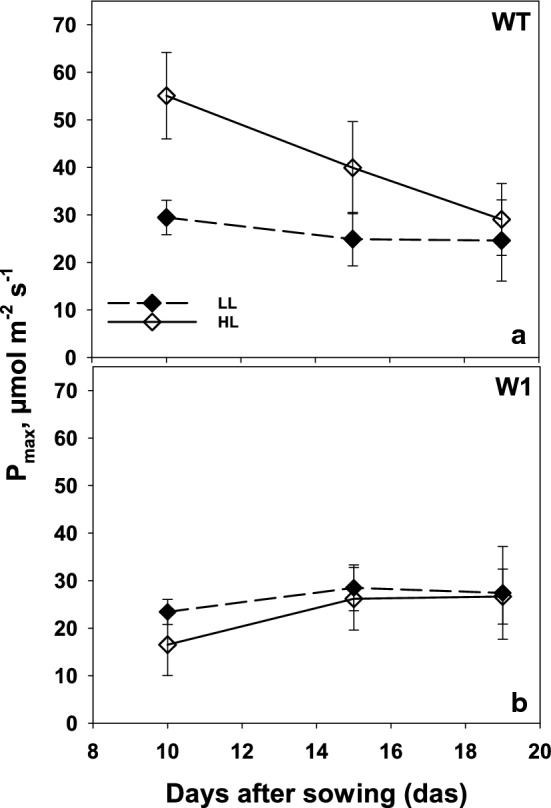
Maximum photosynthesis of primary foliage leaves of LL (filled symbols) and HL (open symbols) grown plants for both WT (**a**) and W1 (**b**) plants as a function of days after sowing in saturating light and at 2000 ppm CO_2_. Depicted values are means ± standard deviation of *n* = 9–15 leaves in total from three independent experiments each comprising 3–5 leaves

Wild-type plants grown under high light showed significantly (three-way ANOVA, Tab. S3, *P* < 0.0001) higher maximum photosynthesis as compared to the low light-grown WT (Fig. 4a). Despite of the significant difference (*P* = 0.001) between HL and LL grown WT plants at day 15, the *P*_max_ values decreased in older HL-grown WT leaves and showed similar values (no significant difference, *P* = 0.999) as LL-grown WT plants at day 19.

In 10-day-old transgenic plants, *P*_max_ values did not differ significantly (*P* = 0.909) between plants grown under low and high light. *P*_max_ had an increasing tendency at days 15 and 19 showing virtually the same values in both groups (Fig. 4b). In addition, there were no significant differences between WT and W1 plants, neither in LL nor in HL (*P* > 0.9999 in both cases) on day 19. However, at days 10 and 15, high light-grown W1 plants showed significantly lower photosynthetic capacity compared to that of WT (*P* < 0.0001 and *P* = 0.0115, respectively). The results show that even after full leaf development, the photosynthetic capacity of WHIRLY1 deficient HL plants never exceeded that of LL-grown WT plants. The larger difference in photosynthetic capacity at early stages of development is likely caused by the delayed chloroplast development which is also obvious by lower *F*_v_/*F*_m_ values (Krupinska et al. 2019).

The differences in the responses of photosynthetic capacity of WT and W1 plants to higher irradiance can be clearly seen in the starch granule formation in their chloroplasts (Fig. S3). Only WT plants from HL conditions showed appreciable starch granules.

#### RubisCO limited photosynthesis

**Fig. 5:**
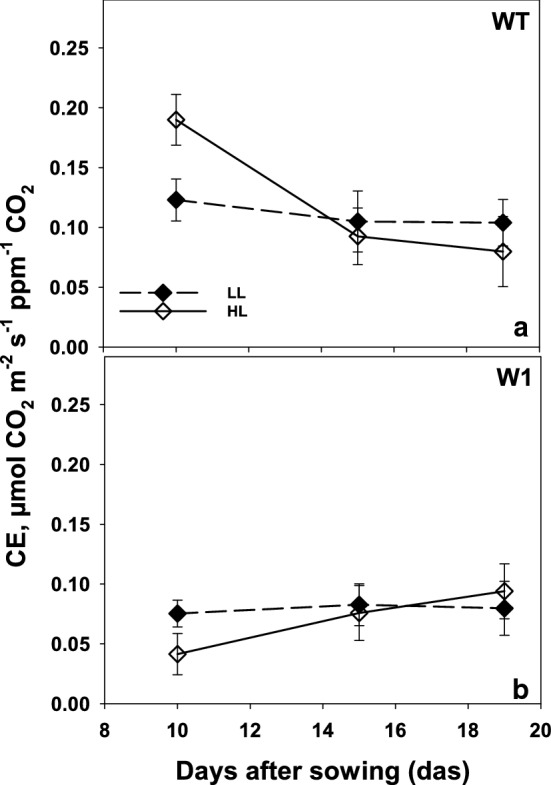
Carboxylation efficiencies (initial slope of *A*/*C*_i_ curve, CE) as a function of days after sowing in LL (filled symbols) and HL (open symbols) grown plants for both WT (**a**) and W1 (**b**). Depicted values are means ± standard deviation of *n* = 9–15 leaves in total from three independent experiments each comprising 3–5 leaves

A similar developmentally affected time course was observed when carboxylation efficiency (CE) was calculated (Fig. 5). CE in high light-grown WT plants was significantly (three-way ANOVA, *P* < 0.0001) higher than in low light-grown WT plants (Fig. 5a). But afterward, CE values declined to values similar to those of low light-grown ones at day 19 (no significant differences, *P* = 0.983 for 15 days and *P* = 0.504 for 19 days).

In WHIRLY1 deficient plants, CE in 10-day-old plants from the high light group was significantly (three-way ANOVA, Tab. S4, *P* = 0.0027) lower than that of low light-grown plants (Fig. 5b). While CE stayed almost constant from day 10 to 19 in low light plants (*P* > 0.9999), these values increased significantly in high light plants from day 10 to day 19 (*P* < 0.0001) to become similar to those in LL plants (*P* = 0.983) (Fig. 5b).

In comparison to the wild type, the WHIRLY1 deficient plants at day 10 showed significantly lower CE values grown under either HL or LL (*P* < 0.0001). No significant differences were detected in CE values of WT and W1 plants at day 19 (*P* = 0.440 for LL and *P* = 0.985 for HL plants). While CE values decreased during the development of wild-type leaves, they slowly increased in the WHIRLY1 deficient plants at HL, but not at LL, and never reached the CE values of WT at day 10.

#### RubisCO abundance

**Fig. 6:**
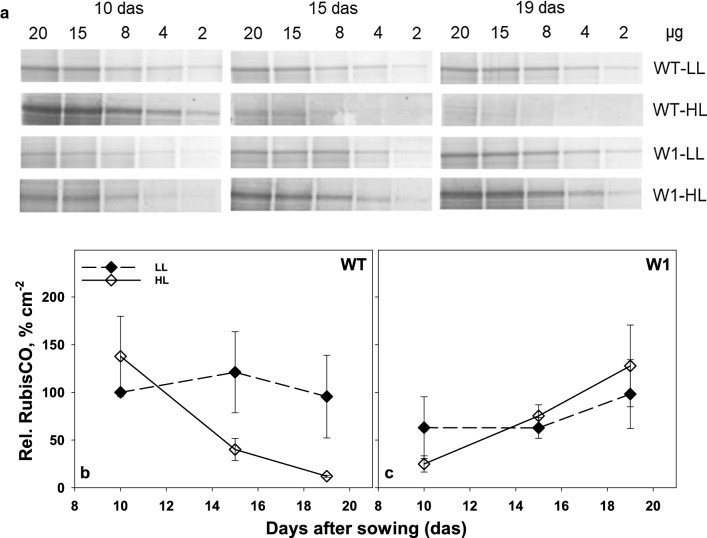
**a** Representative SDS-PAGE from one of three independent experiments showing RbcL bands at different total protein concentrations loaded on the gels. (**b** and **c**) Relative RbcL content per total protein per leaf area derived from SDS-PAGE as a function of days after sowing in WT (**b**) and W1 (**c**) grown under low and high light. Values are expressed in % of the values obtained for LL grown WT leaves at day 10. Depicted values are means ± standard deviation of three independent experiments

To investigate whether the low RubisCO activity of the WHIRLY1 deficient leaves indicated by CE could be a result of a lower amount of RubisCO, RubisCO abundance was analyzed by SDS-PAGE (Fig. 6a). 10-day-old WT plants grown under high light had higher relative RbcL content in comparison to low light grown ones (Fig. 6b). However, in WT plants, the abundance of large subunits of RubisCO in HL-plants decreased strongly from day 10 to day 19, whereas it remained rather stable in low light-grown WT plants during this time course (Fig. 6b).

RubisCO contents in LL-grown W1 plants showed similar levels as in LL-grown WT plants at 10 days. Nevertheless, RubisCO abundance in HL-grown transgenic plants remained slightly lower than in LL-grown ones despite an increase from day 10 to day 19 (Fig. 6c) which ultimately reached to similar values as in 10-day-old WT plants. While RbcL abundance decreased during development in the WT, it stayed almost stable in WHIRLY1 deficient leaves being in accordance with the observation that senescence processes negligibly respond to HL in the WHIRLY1 deficient plants (Kucharewicz et al. 2017).

#### RubisCO abundance and carboxylation efficiency

**Fig. 7:**
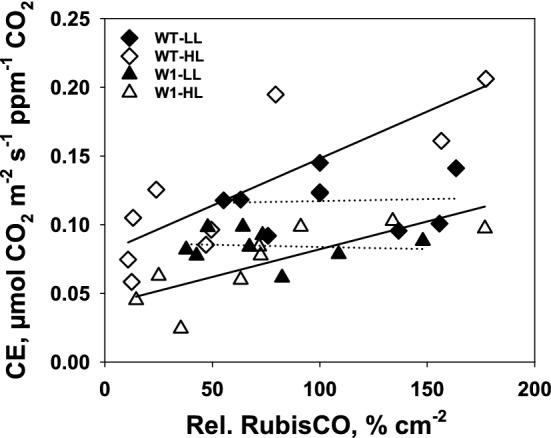
Relationship between carboxylation efficiency (CE) and relative RbcL content per leaf area derived from SDS-PAGE in WT (diamonds) and W1 plants (triangles) growing under low (filled symbols) and high light (open symbols), at different developmental stages. The lines were calculated by regression analysis, where the regressions for the WT and W1 plants growing in low light were not significant, as indicated by the dotted representation

To investigate if carboxylation efficiency was dependent on the RubisCO amount, CE calculated for day 10, 15, and 19 was plotted against the relative RbcL content per leaf area, derived from SDS-PAGE, in the corresponding leaf segments (Fig. 7).

Overall, there was a correlation between RubisCO protein contents and CE in the high light-grown plants. In contrast, the RubisCO amount had no effect on carboxylation efficiency in LL plants. The slopes of the regression lines for the LL-grown plants did not differ significantly from zero (dotted regression lines shown in Fig. 7). However, data points from LL-grown WT leaves were shifted to higher CE values as compared to those of W1 plants. The same was observed for HL-grown plants.

Apparently, CE is not only dependent on the RubisCO abundance, but also on other factors, presumably the activation state of the enzyme.

#### Leaf cross-sections and leaf mass per area (LMA)

Part of high light acclimation of area-based photosynthesis is normally due to increases in leaf thickness (Givnish 1988; Murchie et al. 2005). In bifacial leaves, especially the palisade parenchyma increases (Lichtenthaler et al. 1981). Therefore, leaf anatomy was also investigated to further analyze the reasons for the lack of HL acclimation in W1 plants.

**Fig. 8:**
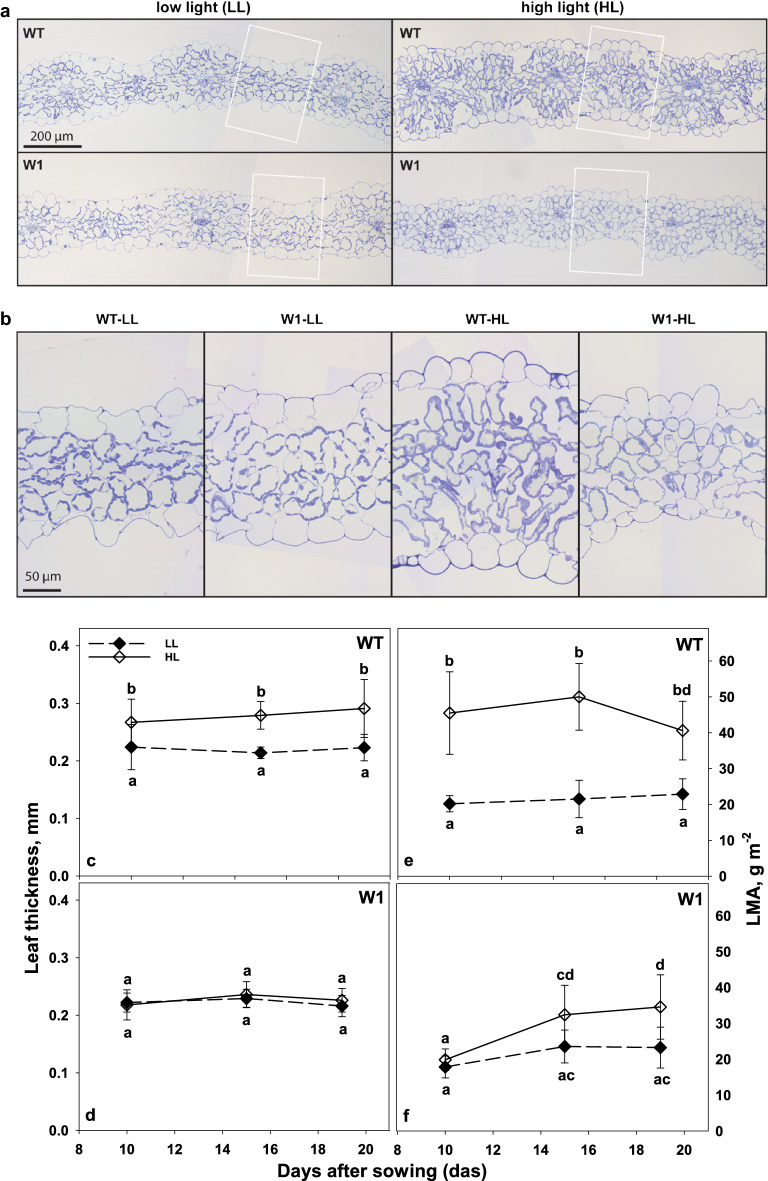
Morphological analysis of WT and W1 primary foliage leaves growing under low light (LL) or high light (HL) conditions (**a**, **b**). Transverse, 500-nm thin sections of leaves embedded in epon were stained with Richardson’s solution. Overview images taken with a ×10 objective (**a**) illustrate the thickness of leaves. Areas indicated with white squares were imaged with a ×40 objective (**b**) and illustrate the shape of mesophyll cells. Thickness and leaf mass per area (LMA) of primary foliage leaves of WT (**c**, **e**) and W1 (**d**, **f**) plants grown at low (filled symbols) and high irradiance (open symbols) as a function of days after sowing. Depicted values are means ± standard deviation of 9–20 leaves in total from three independent experiments each comprising 3–7 leaves. The letters indicate statistically different values at a significance level of *P* = 0.05, as determined by three-way ANOVA, followed by pairwise multiple means comparisons with the Holm–Sidak method

Leaf thickness and mass per area were determined for 15- and 19-day-old plants (Fig. 8). As expected, leaf thickness did not further change after day 10. While W1 leaves from both LL and HL had a thickness not different from that of LL grown WT leaves (Fig. 8c, d) (three-way ANOVA, Tab. S5, *P* > 0.9999 in case of all 3 days), HL-grown WT leaves were about 20% thicker than LL grown leaves (*P* = 0.0002, *P* = 0.0005 and, *P* < 0.0001 for days 10, 15 and, 19, respectively) (Fig. 8c). This was not caused by an increased number of cells but by an increased volume especially of the layers close to the adaxial and abaxial epidermis (Fig. 8a, b).

Wild type plants grown in high light showed significantly higher LMA than in low light (three-way ANOVA, Tab. S6, *P* < 0.0001 for all three days) (Fig. 8e). Moreover, leaves of wild-type plants had a stable leaf mass per area from day 10 to day 19 (Fig. 8e). However, WHIRLY1-deficient transgenic plants showed the same leaf mass per area at days 10 and 15 in both light conditions (*P* = 0.9923 and *P* = 0.0954, respectively, Fig. 8f) with an increasing tendency leading to a significant difference between LL and HL-grown transgenic plants (*P* = 0.007) on day 19.

#### Relative cytoplasmic volume

**Fig. 9:**
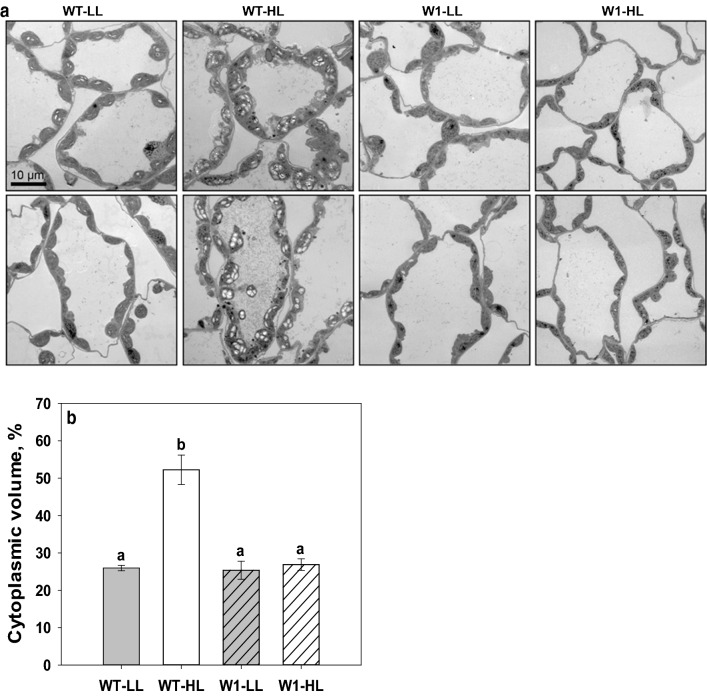
Ultrastructural analysis of mesophyll in primary leaves grown under high or low light for 10 days. **a** Representative transmission electron micrographs of primary leaf mesophyll in WT and W1 plants grown under LL or HL. **b** The cytoplasm to cell volume ratio based on a stereological analysis of electron micrographs shown in **a**. Columns indicate mean ± standard error based on three leaves. For each leaf, between 15 and 23 electron micrographs were analysed. The letters indicate statistically different values at a significance level of *P* = 0.05, as determined by Two-way ANOVA test, followed by pairwise multiple means comparisons with the Holm–Sidak method

In order to elucidate the reasons for the higher leaf mass per area value in WT plants grown at HL, morphological and ultrastructural analyses of leaf mesophyll were performed. No differences in the number of cell layers were observed between the samples when semi-thin resin sections were imaged in a light microscope. Stereological analysis, based on electron micrographs of thin sections (Fig. 9a) however, revealed that the cytoplasm to cell volume ratio was higher in WT plants grown for 10 days under high light compared to low light (two-way ANOVA, Tab. S7, *P* < 0.001) (Fig. 9b). This difference between LL and HL conditions was not observed in the case of the WHIRLY1 deficient plants (*P* = 0.673). Ultrastructural analysis therefore suggests that the relative cytoplasmic volume increased in response to higher irradiance in WT plants, but not in transgenic plants (Fig. 9b).

## Discussion

Since decades, it has been discussed that the photosynthetic apparatus is an environmental sensor involved in acclimation to changes in irradiance (Anderson et al. 1995; Bräutigam et al. 2009; Dietz 2015) resulting in changes in nuclear gene expression (Pogson et al. 2008; Pfannschmidt et al. 2020). Due to its dual localization in chloroplasts and nucleus, WHIRLY1 is an excellent candidate for communication between chloroplasts and nucleus. The results of previous investigations with WHIRLY1 deficient plants (Kucharewicz et al. 2017; Swida-Barteczka et al. 2018) indicated that WHIRLY1 augments the responsiveness of barley to light. It remained an open question whether WHIRLY1 boosts light responses by a specific impact on photosynthesis or whether it has a more general function in plant responses to light. To investigate whether WHIRLY1 affects light acclimation at different levels, besides parameters of photosynthesis also leaf morphology parameters related to high light acclimation have been compared between barley WHIRLY1 deficient plants and wild-type plants in a high light situation requiring acclimation to avoid oxidative stress.

### WHIRLY1 is important for acclimation of photosynthesis to high light

It has been reported before that in WHIRLY1 deficient plants, chloroplast development is delayed (Kucharewicz et al. 2017; Krupinska et al. 2019). According to the leaf chlorophyll contents observed in this study, maturity of W1 leaves was reached after 15 days instead of 10 days as observed in WT plants. Hence, photosynthesis should be compared between the 15-day-old W1 leaves and the 10-day-old wild-type leaves.

Acclimation of plants to high light involves an increase in photosynthesis as measurable as the light saturated CO_2_ fixation rate and carboxylation efficiency (Björkman 1981; Leong and Anderson 1984a, b). Whereas wild-type barley plants showed the expected increase in light saturated rate of photosynthesis, WHIRLY1 deficient plants had no higher photosynthesis in high light compared to low light (Fig. 1). Rather, the CO_2_ assimilation rate was initially lower at high light compared to low light, potentially due to oxidative stress, which had been shown for these plants when they were grown in continuous light of high irradiance (Swida-Barteczka et al. 2018). An important mechanism for adjustment of the capacity of photosynthetic dark reactions to the high rate of delivery of NADPH_2_ and ATP in high light would be an increase in RubisCO concentration and activity (Björkman 1981; Evans 1988). This had been shown for a number of different species using in vitro measurements of RubisCO activity. Indeed, in wild-type leaves at day 10, the relative amount of the large subunit of RubisCO (RbcL) at high light was higher than at low light as shown by SDS-PAGE analyses (Fig. 6). With increasing age, *P*_max_, CE and RbcL content declined strongly in WT leaves at HL, presumably because of high light-promoted premature senescence, which was apparent by the decline of chlorophyll content after day 15 (Fig. 3). A decrease of the RubisCO content accompanied by a decline in photosynthesis after the leaves have reached full expansion has been reported for many grasses (Mae et al. 1983; Makino et al. 1984; Suzuki et al. 2010). Also in rice, an age-related decline of photosynthetic capacity (*P*_max_) together with a decrease of RubisCO content was reported to be accelerated under high light (Makino et al. 1985; Hidema et al. 1991, 1992; Murchie et al. 2002). The decline in *P*_max_ and RubisCO content in plants growing under high irradiance was shown to begin after full leaf expansion (Evans 1983; Suzuki et al. 2009) or 3–4 days after reaching the maximum values (Murchie et al. 2002). While in the barley wild type, photosynthesis tended to decline with increasing age of the fully expanded leaves, it increased with age in the also fully expanded W1 leaves similarly in LL and HL conditions. This increase in photosynthesis was accompanied by an increase in chlorophyll content from day 10 on, leveling off after day 15. Despite the different kinetics, the data on photosynthesis clearly show that WHIRLY1 deficient plants lack the typical growth irradiance dependent differences in maximum photosynthesis and carboxylation efficiencies. Rather, the missing high light acclimation seems to have caused additional problems as photosynthesis rates of HL plants tended initially to stay below those of LL plants.

To investigate RubisCO activity in vivo, photosynthesis was measured at intercellular CO_2_ concentrations below 200 ppm (Long and Bernacchi 2003; Lombardozzi et al. 2018). The slope of the A/C_i_ curve in this range, the so-called carboxylation efficiency (CE), in W1 plants was strongly reduced and did not respond to higher growth irradiance (Fig. 5). In contrast, WT leaves displayed an increased CE at HL, in parallel to higher amounts of RbcL per area. Hence, WT leaves were able to acclimate RubisCO activity to high light, whereas W1 leaves were not.

A comparison of CE and RbcL content might indicate how acclimation may have been achieved. In WT leaves at high light, a correlation between CE and RbcL could be observed (Fig. 7). Still considerable photosynthesis was observed in the presence of an apparently very low level of RbcL that cannot be properly quantified by colloidal Coomassie Blue staining of gels. Nevertheless, at HL, the CE of WT plants seemed to be at least partially dependent on RbcL content. While in the WT, the relationship between both parameters was altered by a senescence related decline during the observation period, in W1 leaves during the same period, the relationship between the two parameters was affected by an increase in photosynthetic activity. Also here, a good correlation between both parameters was observed, albeit at a much lower level of CE. In general, the overall range of the observed RbcL amounts were similar in both genotypes and both light conditions, indicating that carboxylation activity in vivo, as reflected by CE, was to a lesser extent governed by the amount of enzyme, but rather by an additional factor, presumably the activation state of RubisCO. These results suggest that carboxylation efficiency is a function of different parameters like age and developmental stage rather than only the amount of RubisCO in the leaf. Moreover, the results suggest that RubisCO activation is somehow controlled by the abundance of WHIRLY1.

### WHIRLY1 is also required for light acclimation of leaf morphology

Besides the biochemical adjustments in chloroplast function, acclimation to light involves also changes in the anatomy of leaves (Givnish 1988; Lichtenthaler et al. 1981). Increased photosynthetic capacity in high light acclimated plants is followed by a higher carbon and nitrogen investment into RubisCO together with leaf structural changes in order to enable a faster rate of gas exchange (Seemann et al. 1987; Murchie and Horton 1997; Oguchi et al. 2003).

While wild-type leaves followed the expected trend and were thicker when grown at high light compared to low light, leaves of WHIRLY1 deficient plants did not show a light dependent change in thickness or leaf mass per area (Fig. 8). In many species, sun leaves contain more palisade layers and larger palisade cells than shade leaves (e.g., Lichtenthaler et al. 1981). Thicker leaves in high light acclimated plants of *Chenopodium album* L. were reported to be mainly caused by an elongation of palisade cells or an increase in the number of palisade cell layers (Yano and Terashima 2001, 2004). However, when leaf sections of wild-type barley and WHIRLY1 deficient plants were compared after growth at different irradiances, no difference was detected in the number of cell layers (Fig. 8a and b). Also, the thicker leaves of high light-grown rice did not differ from low light-grown rice with respect to cell number (Murchie et al. 2005). It is likely, that dicotyledonous and monocotyledonous plants differ in the strategies to increase leaf thickness in high light. Histological analyses of images obtained from ultrathin leaf sections revealed that in the mesophyll of WT leaves from high light-acclimated plants the ratio of cytoplasm/cell volume was twice as high as in leaves of low light-grown plants (Fig. 9). In contrast, in the leaves of the WHIRLY1 deficient plants this ratio did not change in response to irradiance. Organelles in the cytoplasm presumably have a higher density than the vacuole (Poorter et al. 2009). Therefore, the higher LMA of high light-grown WT leaves may be caused mainly by the higher cytoplasm/cell volume ratio. In addition, the higher starch content of these leaves may also contribute to the high LMA. Poorter et al. (2009) reported that not leaf volume per area but rather the leaf density explains LMA in a large number of species, including grasses. Together with the reduced thickness of leaves, the low values obtained for the leaf mass per area (LMA) indicate that the W1 plants even at high irradiance have leaves morphologically resembling shade leaves.

The lack of acclimation to high irradiance of both photosynthesis and leaf morphology indicate that W1 plants are unable to properly respond to light. This suggests that WHIRLY1 might be required either for an efficient functionality of the photosensory systems such as phytochromes and/or signal transduction processes required for the appropriate responses controlled by these systems. Investigations with plants overexpressing *PHYTOCHROME B* (*PHY-B)* suggest that light acclimation of both the photosynthetic apparatus and leaf morphology are controlled by photoreceptors such as PHY-B (Kreslavski et al. 2018). However, in an Arabidopsis mutant lacking PHY-B, photosynthetic acclimation has been shown to respond to light (Walters et al. 1999). Therefore, besides photoreceptors, further light-measuring mechanisms might be involved in light acclimation.

Indeed, development of chloroplasts and of leaf photosynthetic structures were reported to be also controlled by light that is perceived by the chloroplasts themselves (Lepistö and Rintamäki 2012). While the impact of light on leaf thickness is an early event in leaf development occurring before leaf expansion has been stopped (Poorter et al. 2009), chloroplast differentiation and photosynthesis can be still dynamically adjusted to the local light environment in fully expanded leaves (Yano and Terashima 2001). In the WHIRLY1 deficient barley plants, light acclimation is obviously impaired at the two levels, i.e. the level of leaf morphology determined at early development and at the level of chloroplast operation in fully developed leaves. This hints at a coordinative role of WHIRLY1 in translating information about the light environment at early plant development into adjustments of chloroplast structure and the photosynthetic apparatus that match the preceding early light dependent adjustments in leaf morphology. A role of WHIRLY1 in coordinating the light response at early leaf development is in accordance with its high abundance in undifferentiated cells at the base of primary foliage leaves (Krupinska et al. 2014). This systemic effect of WHIRLY1 on leaves and chloroplasts makes sense, as the plant cannot perform efficient photosynthesis without having the appropriate leaf morphology. How WHIRLY1 mediates coordination of the processes at the molecular level remains to be investigated.

The results of this study are in accordance with the findings of previous investigations performed with WHIRLY1 deficient barley plants (Kucharewicz et al. 2017; Swida-Barteczka et al. 2018). Whereas the impact of WHIRLY1 on senescence (Kucharewicz et al. 2017) and photosynthesis mediated ROS production at high irradiance (Swida-Barteczka et al. 2018) could be the consequence of structural changes in chloroplasts caused by the chloroplast nucleoid associated WHIRLY1, this study shows that the impact of WHIRLY1 on the responsiveness of plants to light is more comprehensive comprising the two levels of leaf morphology and chloroplast functionality. While a low level of WHIRLY1 in barley is obviously sufficient to enable light dependent chloroplast development, albeit at reduced rate, the higher abundance in the wild type is required for coordinated light acclimation at different levels.

#### *Author contribution statement*

MS, WB and KK conceived and designed research. Material preparation, conduction of experiments and data analysis were performed by MS. Transmission electron microscopy and leaf cross section was done by UR. The first draft of the manuscript was written by MS. All authors commented and revised the previous versions of the manuscript. All authors read and approved the final manuscript.

## Supplementary Information

Below is the link to the electronic supplementary material.

Supplementary file1 (PDF 181 KB)

## Data Availability

The data that support the findings of in this study are available in the Supplementary Information of this article. The raw datasets in this study are available from the first author or corresponding author on reasonable request.
