# Supplementary material for: The plastid-nucleus localized DNA-binding protein WHIRLY1 is required for acclimation of barley leaves to high light
Source: Planta. 2022 Mar 13;255(4):84. doi: 10.1007/s00425-022-03854-x (PMC8918454; doi:10.1007/s00425-022-03854-x)
Supplement: Supplementary file 1 — Supplementary file1 (PDF 181 KB) [file 425_2022_3854_MOESM1_ESM.pdf]

The plastid-nucleus localized DNA-binding protein WHIRLY1 is required for acclimation of barley leaves to high light

Monireh Saeid-Nia[1], Urska Repnik[2], Karin Krupinska[1], Wolfgang Bilger[1]

[1] Institute of Botany, Christian-Albrechts-University, Kiel, Germany

[2] Central Microscopy, Department of Biology, Christian-Albrechts-University, Kiel, Germany

Supplementary Data

Tab. S1 Two-way ANOVA analysis of variance for CO_2 assimilation rate at 1500 µmol m^{-2} s^{-1} and in presence of 380 ppm CO_2 in WT and W1 plants grown under either low light or high light and at 10 days after sowing

Source of Variation	DF	SS	MS	F	P
Factor 1: genotype	1	2358.512	2358.512	152.285	<0.001
Factor 2: light	1	16.242	16.242	1.049	0.313
Factor 1 x Factor 2	1	419.602	419.602	27.093	<0.001
Residual	33	511.086	15.487		
Total	36	3409.465	94.707		

Tab. S2 Three-way ANOVA analysis of variance for chlorophyll content for WT and W1 plants grown under low and high light at different ages (10, 15, and 19 days after sowing)

Source of Variation	DF	SS	MS	F	P
Factor 1: genotype	1	11556.472	11556.472	332.963	<0.001
Factor 2: light	1	3417.565	3417.565	98.466	<0.001
Factor 3: age	2	2404.694	1202.347	34.642	<0.001
Factor 1 x Factor 2	1	511.749	511.749	14.744	<0.001
Factor 1 x Factor 3	2	3864.964	1932.482	55.678	<0.001
Factor 2 x Factor 3	2	648.224	324.112	9.338	<0.001
Factor 1 x Factor 2 x Factor 3	2	1362.243	681.122	19.624	<0.001
Residual	344	11939.528	34.708		
Total	355	35978.175	101.347		

Tab. S3 Three-way ANOVA analysis of variance for CO_2 assimilation rate at saturating light and in presence of 2000 ppm CO_2 (P_{max}) in WT and W1 plants grown under either low light or high light and at different ages (10, 15, and 19 days after sowing)

Source of Variation	DF	SS	MS	F	P
Factor 1: genotype	1	1937.423	1937.423	35.580	<0.001
Factor 2: growth light	1	805.912	805.912	16.048	<0.001
Factor 3: age	2	283.213	141.608	2.820	0.065
Factor 1 x Factor 2	1	1991.606	1991.606	39.659	<0.001
Factor 1 x Factor 3	2	2237.461	1118.730	22.277	<0.001
Factor 2 x Factor 3	2	224.221	112.111	2.232	0.114
Factor 1 x Factor 2 x Factor 3	2	740.616	370.308	7.374	0.001
Residual	84	4218.352	50.218		
Total	95	13688.675	144.091		

Tab. S4 Three-way ANOVA analysis of variance for carboxylation efficiencies in WT and W1 plants grown under low light or high light and at different ages (10, 15, and 19 days after sowing)

Source of Variation	DF	SS	MS	F	P
Factor 1: genotype	1	0.0510	0.0510	118.196	<0.001
Factor 2: growth light	1	0.0000152	0.0000152	0.0353	0.851
Factor 3: age	2	0.0102	0.00508	11.782	<0.001
Factor 1 x Factor 2	1	0.00277	0.00277	6.415	0.013
Factor 1 x Factor 3	2	0.0553	0.0276	64.095	<0.001
Factor 2 x Factor 3	2	0.00465	0.00233	5.392	0.006
Factor 1 x Factor 2 x Factor 3	2	0.0287	0.0144	33.309	<0.001
Residual	116	0.0500	0.000431		
Total	127	0.215	0.00196		

Tab. S5 Three-way ANOVA analysis of variance for leaf thickness per area for WT and W1 plants grown under low and high light at different ages (10, 15, and 19 days after sowing)

Source of Variation	DF	SS	MS	F	P
Factor 1: genotype	1	0.0272	0.0272	33.985	<0.001
Factor 2: light	1	0.0344	0.0344	42.888	<0.001
Factor 3: age	2	0.00274	0.00137	1.708	0.184
Factor 1 x Factor 2	1	0.0250	0.0250	31.214	<0.001
Factor 1 x Factor 3	2	0.00164	0.000820	1.024	0.361
Factor 2 x Factor 3	2	0.00288	0.00144	1.795	0.169
Factor 1 x Factor 2 x Factor 3	2	0.000255	0.000127	0.159	0.853
Residual	161	0.129	0.000801		
Total	172	0.226	0.00131		

Tab. S6 Three-way ANOVA analysis of variance for leaf mass per area for WT and W1 plants grown under low and high light at different ages (10, 15, and 19 days after sowing)

Source of Variation	DF	SS	MS	F	P
Factor 1: genotype	1	2368.336	2368.336	53.162	<0.001
Factor 2: light	1	8540.683	8540.683	191.712	<0.001
Factor 3: age	2	1107.254	553.627	12.427	<0.001
Factor 1 x Factor 2	1	2364.286	2364.286	53.071	<0.001
Factor 1 x Factor 3	2	852.983	426.492	9.573	<0.001
Factor 2 x Factor 3	2	166.205	83.102	1.865	0.159
Factor 1 x Factor 2 x Factor 3	2	475.722	237.861	5.339	0.006
Residual	143	6370.582	44.550		
Total	154	24487.535	159.010		

Tab. S7 Two-way analysis of variance for cytoplasm/cell volume of WT and W1 plants grown under low and high light at 10 days after sowing

Source of Variation	DF	SS	MS	F	P
Factor 1: genotype	1	505.772	505.772	27.913	<0.001
Factor 2: light	1	580.762	580.762	32.051	<0.001
Factor 1 x Factor 2	1	460.563	460.563	25.418	<0.001
Residual	8	144.957	18.120		
Total	11	1692.054	153.823		

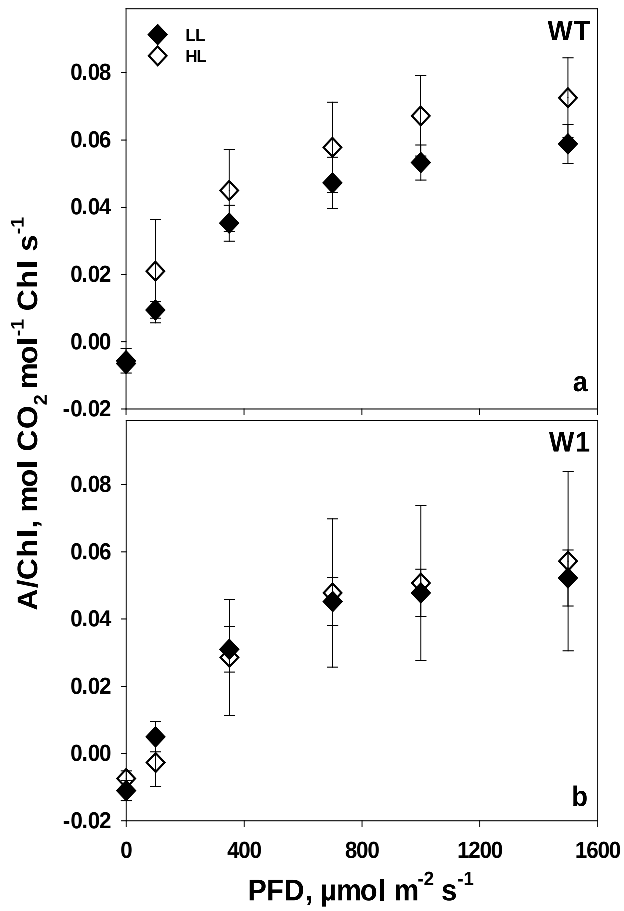

Fig. S1 Light dependency of the CO_2 assimilation rate per chlorophyll content (A/Chl) as a function of incident irradiance (PFD) measured in the presence of 380 ppm CO_2 in LL (filled symbols) and HL (open symbols) grown plants for both WT (a) and W1 (b) at day 10. Depicted are means ± standard deviation of n=9-15 leaves in total from three independent experiments each comprising 3-5 leaves

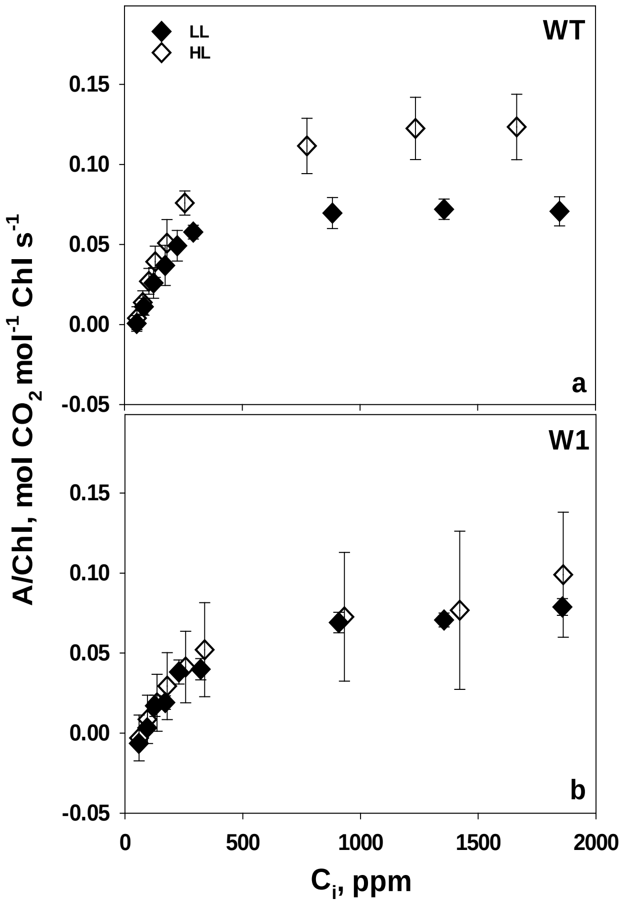

Fig. S2 A per chl content (A/Chl) as a function of internal CO_2 concentration (C_i) measured in LL (filled symbols) and HL (open symbols) grown plants for both WT (a) and W1 (b) at day 10 with 1000-1500 µmol m^{-2} s^{-1} light as the saturating light. Depicted are means ± standard deviation of n=9-15 leaves in total from three independent experiments each comprising 3-5 leaves

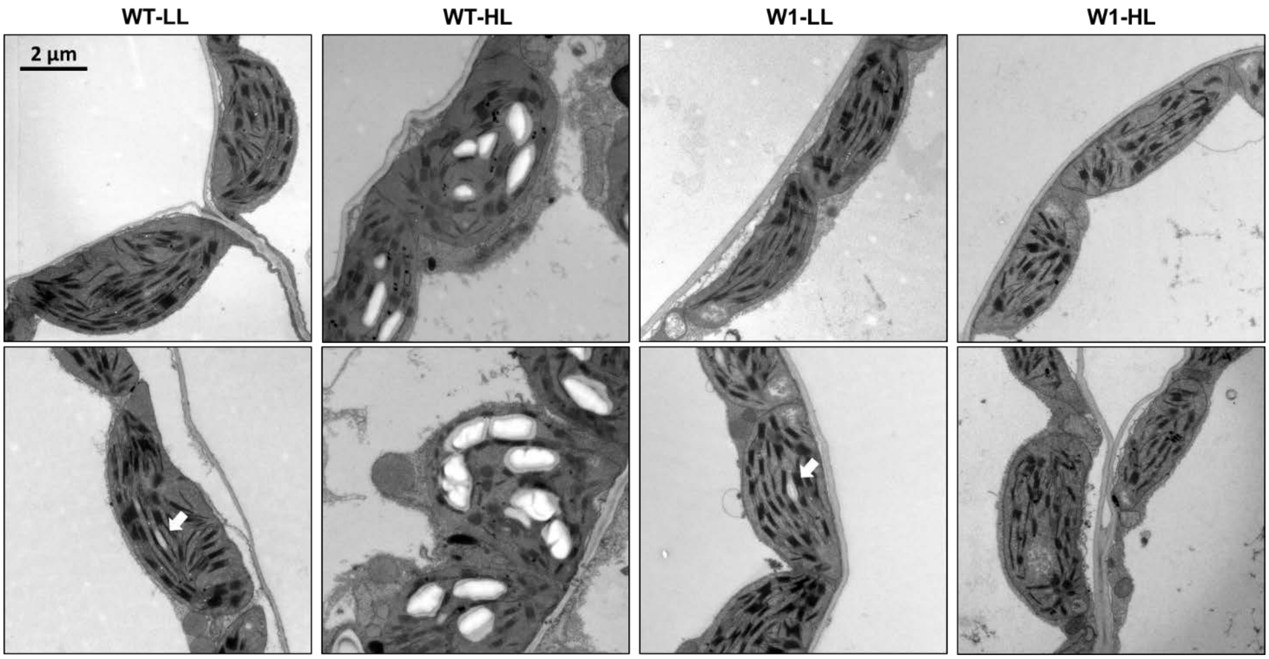

Fig. S3 Ultrastructural analysis of mesophyll in WT and W1 primary leaves grown under high or low light for 10 days, showing differences in the abundance of starch granules in chloroplasts. Two representative electron micrographs are shown for each sample group. Chloroplasts of WT plants grown under high light contain large starch granules, which appear as electron lucent inclusions. In WT and W1 plants grown under low light, occasionally small starch granules are present (white block arrows)
